# *aspA* promoter as a tumor microenvironment-responsive autoswitch for anticancer agent expression in *Escherichia coli*

**DOI:** 10.1186/s13036-026-00743-9

**Published:** 2026-07-31

**Authors:** Dogeun Lee, Seyeon Hong, Jihyeon Kim, Miryoung Song, Daejin Lim

**Affiliations:** 1 Division of Biomedical Convergence, College of Biomedical Science, Kangwon National University, Chuncheon, 24341 Republic of Korea; 2 Department of Bioscience and Biotechnology, Hankuk University of Foreign Studies, Yongin, 17035 Republic of Korea

**Keywords:** *E. coli*, Fumarate respiration, *aspA* promoter, Tumor-specific expression, Tumor microenvironment, Nutrient heterogeneity

## Abstract

Bacterial cancer therapy has emerged as a promising strategy for the local delivery of anticancer agents to solid tumors because bacteria preferentially colonize the tumor microenvironment (TME). However, strategies for exploiting metabolic features of the TME to achieve tumor-selective expression of anticancer agents remain limited. Here, we present a tumor-responsive gene expression system in *Escherichia coli* for tumor-selective expression of the recombinant immunotoxin psp-TGFa-PE38 (TP), exploiting its intrinsic ability to sense metabolic cues in the TME. To identify such TME-associated metabolic cues, we examined tumor metabolites in CT26 tumor-bearing mice and confirmed that tumors contained > 3-fold lower glucose and > 5-fold higher fumarate levels than those in the liver. In response to this nutrient heterogeneity, *E. coli* upregulated genes involved in fumarate respiration, including *frdA*, *dctA*, *dcuB*, and *aspA*, in the DcuSR regulon. *aspA* was most upregulated (65-fold) in tumors, which is indicative of the strongest activity of its promoter in the TME. We used the *aspA* promoter (P_aspA_) as a tumor-specific switch to control TP expression, targeting secreted functional TP and inducing cytotoxicity in vitro. TP was specifically expressed by P_aspA_ in tumors, reducing the tumor size by 2.8-fold and extending survival by 35 days in vivo. Overall, these results suggest that the potential of P_aspA_ as a tumor-specific expression system.

**Supplementary Information:**

The online version contains supplementary material available at https://doi.org/10.1186/s13036-026-00743-9.

## Graphical Abstract

**Figure:**
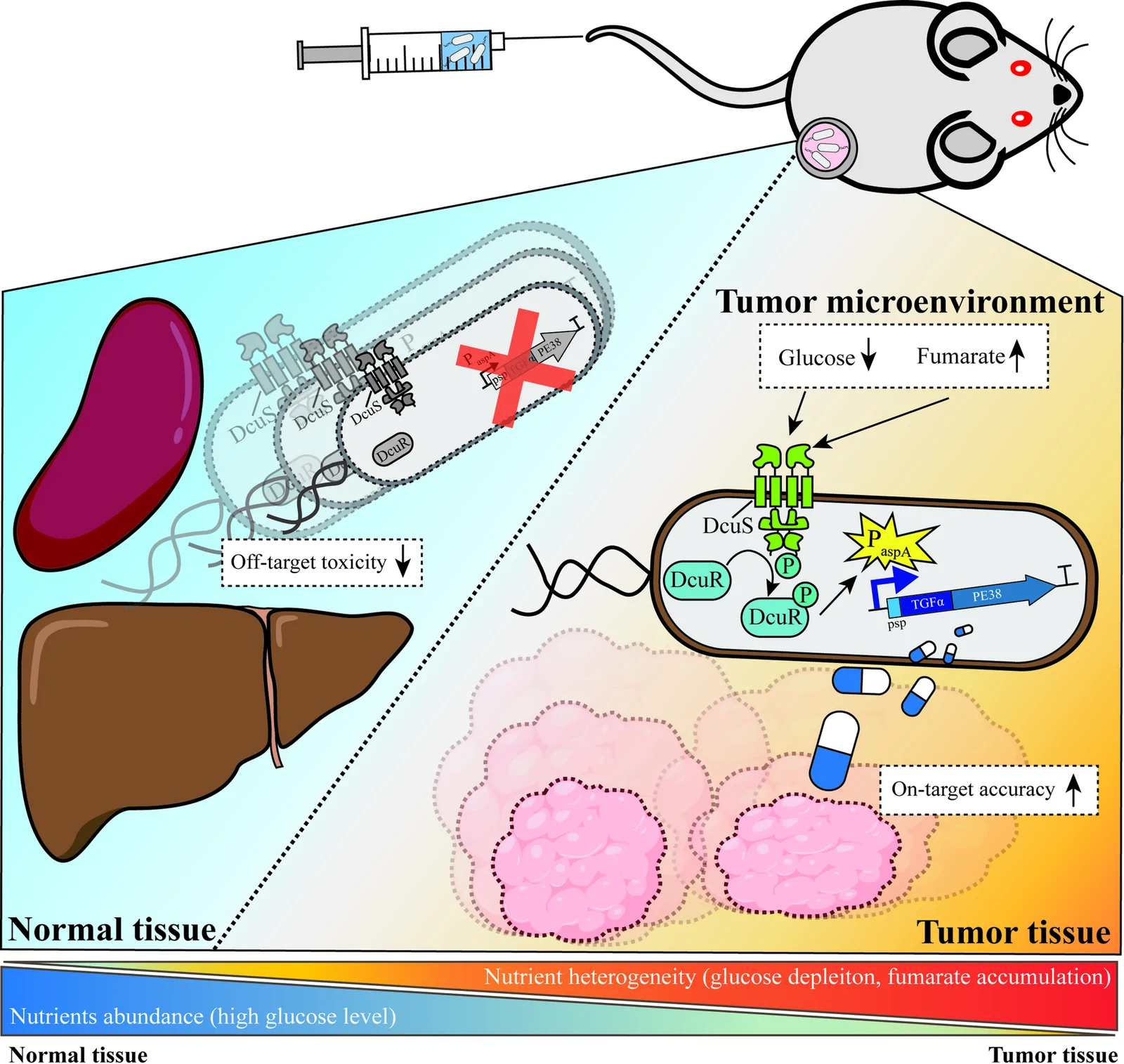

## Introduction

Recent advances in cancer therapy have led to the development of targeted therapies and immunotherapies that aim to minimize the off-target side effects associated with conventional approaches, such as surgery, chemotherapy, and radiotherapy. Although these modalities provide improved selectivity, they often exhibit limited efficacy in solid tumors due to poor tissue penetration. A critical factor in this limitation is the presence of metabolic barriers within the tumor microenvironment (TME), which restricts therapeutic access and fosters an immunosuppressive milieu that hinders anticancer immune responses. Although these features are associated with reduced therapeutic efficacy in solid tumor, they may create a selective niche for bacterial colonization. This unique property has made bacterial cancer therapy and attractive therapeutic strategy [1, 2]. The concept of using bacteria for cancer treatment dates back to the late nineteenth century, when William B. Coley first used bacterial preparations in patients with malignant disease [3]. With advances in genetic manipulation, facultative anaerobes, such as *Escherichia coli*, have emerged as versatile vehicles for tumor-targeted delivery of diverse payloads, including small molecules, immunotoxins, immunomodulators, prodrug-converting enzymes, siRNAs, and nanobodies, to the TME [4–7]. Although bacteria have been widely explored as carriers for tumor-targeted delivery of therapeutic payloads, ensuring that these agents are expressed exclusively within the tumor tissues, without harming normal tissues, remains a major challenge. Constitutive expression of anticancer agents by bacteria may cause off-target toxicity in healthy tissues, highlighting the need for precise control over therapeutic gene expression. Inducible systems may help in overcoming this issue. However, such systems rely on repeated administration of external inducers, which can complicate treatment regimens. This limitation prompted us to investigate whether metabolic features that distinguish the TME from normal tissues could be harnessed as endogenous signals for tumor-specific gene expression. Such regulation is important considering the fact that constitutive expression of anticancer agents by bacteria may damage healthy tissues [8–10].

We hypothesized that metabolic features of the TME could be exploited as endogenous signals for tumor-specific regulation of the expression of anticancer agent. The TME exhibits a metabolic profile that is distinct from that of normal tissues [11]. One of the key differences is the increased rate of glucose uptake by tumor cells, even under normoxic conditions, which was first observed by Otto Warburg and is now known as the Warburg effect [12, 13]. As tumors grow, these heightened metabolic demands, coupled with abnormal vascular architecture and disrupted tissue structure, aggravate nutrient insufficiency within the solid tumor tissues [14]. Unlike normal blood vessels, tumor vasculature branches irregularly, resulting in poor nutrient delivery and considerable heterogeneity in nutrient distribution within the TME [15]. This abnormal vascular architecture, combined with the metabolic reprogramming that is inherent to tumor cells, contributes to the accumulation of oncometabolites, such as succinate and fumarate [16–23]. The TME exhibits characteristic metabolic alterations, including low pH, hypoxia, glucose depletion, and oncometabolite accumulation. These features contribute to an immunosuppressive niche that undermines the effectiveness of conventional anticancer therapies. However, they also generate distinctive environmental cues that can be sensed by bacterial regulatory systems [5, 24].

We hypothesized that intrinsic bacterial sensing systems can detect and respond to tumor-associated environmental cues, such as nutrient heterogeneity. These systems have the potential to enable tumor-specific regulation of gene expression in response to the TME. In *E. coli*, adaptation of nutrient-limited or anaerobic environments involves activation of fumarate respiration, which is a key metabolic pathway that allows utilization of alternative carbon sources such as C_4_-dicarboxylate when glucose and oxygen are scarce [25]. This process requires coordinated expression of genes involved in C_4_-dicarboxylate transport and fumarate reduction, such as *aspA*, *frdABCD*, *dcuA*, and *dcuB*, which are transcriptionally regulated by the DcuSR two-component system that enables *E. coli* to sense and respond to environmental changes in carbon availability under anaerobic conditions [26–32].

Promoters controlled by such a regulatory network can serve as precise biological switches, activating gene expression only under specific environmental condition. In this study, we aimed to develop an expression system in *E. coli* that exploits native metabolic sensing mechanisms responsive to tumor-specific nutrient cues, enabling selective induction of therapeutic protein expression within the TME.

## Materials and methods

### Bacterial strains, plasmid construction, and culture conditions

Table 1 lists the bacterial strains and plasmids used in this study. *E. coli* K-12 MG1655 was used as the wild-type strain and was cultured in Luria–Bertani broth (LB, Cat. No. MB-L4488; MB Cell). To monitor the *aspA* promoter (P_aspA_) activity, a P_aspA_:: green fluorescent protein variant OVA (*gfpOVA*) plasmid (pA-gfpOVA) was constructed using restriction enzyme-based cloning, as follows: first, genomic DNA extracted from *E. coli* K-12 MG1655 was used to amplify the *aspA* promoter region, which included DcuR-dependent binding sites located at positions − 67 to − 87 and − 169 to − 153 relative to the transcription start site [33]. The amplified promoter was cloned into a reporter gene (*gfpOVA*) by replacing the *iroB* promoter sequence with the piroB::gfpOVA plasmid [34] using the specific primer set listed in Supplementary Table S1. To confirm the expression of the anticancer agent, the *araBAD* promoter in the psp-TGF⍺-PE38 plasmid was replaced with P_aspA_ by Gibson assembly using the primer set listed in Supplementary Table S1, consequently generating the P_aspA_::psp-TGF⍺-PE38 plasmid (pA-TP). The plasmids were confirmed by DNA sequencing (Macrogen). Next, each plasmid was transformed into *E. coli* via heat shock. Bacterial strains carrying the plasmid were cultured in LB medium at 37 °C with shaking at 200 rpm. Ampicillin (100 µg/mL, Cat. No. A9518; Sigma-Aldrich) was then added to the solution.

**Table 1:** Bacterial strains and plasmids used in this study

Strains	Description	References
***Escherichia coli*** **K-12 substr. MG1655**		
MG1655	Wild-type *E. coli* MG1655	ATCC
MDG001	*E. coli* carrying pA-gfpOVA	This study
MDG003	*E. coli* carrying pA-TP	This study
**Plasmid**		
pA-gfpOVA	gfpOVA was controlled by P_aspA_ in pBR322	This study
pA-TP	TP was controlled by P_aspA_ in pBR322	This study

Bacterial cells were grown in M9 medium (Cat. No. MM003-01; WELGENE) supplemented with casamino acid hydrolysate (0.1% w/v, Cat. No. MB-C1656; MB cell) and L-tryptophan (0.005% w/v, Cat. No. MB-T4863; MB Cell) [35]. Incubation was performed in an anaerobic bag (Cat. No. KS-B2002; Kisanbio), and the M9 medium was supplemented with 0.4% glucose (Cat. No. 50-99-7; Duksan) or 20 mM fumarate (Cat. No. A10976.36; Thermo Fisher Scientific) [36].

### Fluorescence intensity analysis

Carbon source-dependent P_aspA_ activity was assessed using green fluorescent protein expression within the bacterial cell culture medium, and fluorescence intensity was measured hourly using a spectrophotometer (VLBL00D0; Thermo Fisher Scientific). Measurements were conducted at excitation and emission wavelengths of 488 and 513 nm, respectively, to detect gfpOVA accumulation. The absorbance was measured at 600 nm to standardize the fluorescence intensity for each group.

### Western blot analysis

To examine the expression of TP by P_aspA_ in vitro, MG1655 cells carrying pA-TP were cultured in M9 medium with restrictively supplemented carbon sources for 24 h. Whole bacterial cells, pellets, and supernatants were collected at the indicated time points. Bacterial supernatants were filtered through a 0.2-micrometer filter (Cat. No. FJ13ASCCA002DL01; GVS Filter), mixed with 5× SDS, and boiled at 100 °C for 5 min. To confirm TP expression in *vivo*, MDG003 (1 × 10^8^ CFU/mouse) was injected into mice grafted with CT26 tumors through the tail vein when the tumors reached a volume of 100–130 mm^3^. Tumors and livers were excised at the indicated time points and homogenized in 1 mL of RIPA buffer (Cat. No. IBS-BR002 Intron Biotechnology) containing 1× Protease & Phosphatase Inhibitor Cocktail and 1× EDTA (Cat. No. 78440; Thermo Fisher Scientific). The filtered supernatants were mixed with 5× SDS and boiled at 100 °C for 10 min. Total proteins were separated via electrophoresis on a 10% sodium dodecyl sulfate-polyacrylamide gel and transferred onto nitrocellulose membranes (Cat. No. IPVH00010; Sigma-Aldrich). The membrane was blocked with 5% skim milk for 1 h and probed with rabbit anti-*Pseudomonas* exotoxin A antibody (Cat. No. P2318; Sigma-Aldrich) or mouse anti-ß-actin antibody (Cat. No. sc-47778; Santa Cruz Biotechnology) overnight at 4 °C. After washing off the primary antibodies with TBS (140 mM NaCl, 10 mM Tris-HCl; pH 8.0) containing 0.1% Tween 20, the membranes were incubated at room temperature (20 ± 5 °C) for 1 h with goat anti-rabbit IgG (Cat. No. 7074s; Cell Signaling Technology) and horse anti-mouse IgG (Cat. No. 7076s; Cell Signaling Technology) conjugated to horseradish peroxidase. The bound proteins were visualized using an ECL kit (Cat. No. 32209; Thermo Fisher Scientific).

### Cell lines and culture

CT26 murine colon carcinoma, MC38 murine colon carcinoma, 4T1 murine mammary carcinoma, LLC1 lung carcinoma, and SW620 human colon carcinoma cells were purchased from the American Type Culture Collection. CT26, MC38, 4T1, and LLC1 cells were grown in high-glucose Dulbecco’s modified Eagle medium (Cat. No. AL007A; HIMEDIA) containing 10% fetal bovine serum (FBS, Cat. No. SH30919.03; cytiva HyClone™) and 1% penicillin-streptomycin (Cat. No. 30-002CI; Corning Inc.), whereas SW620 cells were grown in Roswell Park Memorial Institute 1640 (Cat. No. AL028A; RPMI, HIMEDIA) medium supplemented with 10% fetal bovine serum and 1% penicillin-streptomycin.

### Animal ethics and experiments

All animal procedures were performed in accordance with the National Guidelines for the Care and Use of Laboratory Animals and the ARRIVE (Animal Research: Reporting of In Vivo Experiments) guidelines were approved by the Institutional Animal Care and Use Committee (IACUC) of Kangwon National University (approval number KW-240326-3). Female BALB/c mice (5–6-week-old; body weight, 18–20 g) were purchased from Samtako Bio Company. Mice were maintained under specific-pathogen-free conditions in a biosafety level 2 facility with a 12-hour light/dark cycle, controlled temperature (22 ± 2 °C), and free access to standard chow and water. After an acclimation period of at least 7 days, mice were randomly assigned to experimental groups. Mice carrying subcutaneous tumors were generated as follows: tumor cells cultured in vitro were harvested, suspended in 30 µL phosphate-buffered saline (PBS), and injected subcutaneously into the right thigh at the indicated dose (1 × 10^6^ cells for CT26). Bacterial injections were administered when the tumors reached a volume of 100–150 mm^3^. To evaluate the antitumor effects of TP immunotoxin, mice grafted with CT26 cells were injected with wild-type *E. coli* or *E. coli* carrying pA-TP through the tail vein. The tumor volume was calculated using the formula: (*L* $\:\times\:\:$*H* $\:\times\:$ *W*)/2, where L is the length, W is the width, and H is the height of the tumor in millimeters. For bacterial distribution in vivo, solid tumors and other organs were extracted from mice and homogenized in 1×PBS using a homogenizer (Ultra–Turrax T10; IKA). Mice with tumor volumes ≥1,500 mm^3^ were humanely euthanized using a CO_2_ chamber, in accordance with the guidelines of the Animal Research Committee of Kangwon National University. The survival rates were estimated using the Kaplan–Meier curve.

### Assessment of cell viability

To evaluate the cytotoxic effects of TP on cancer cells, cell viability was assessed using an MTT assay kit (Cat. No. M3353; Tokyo Chemical Industry) after exposure to bacterial culture supernatant. Briefly, the bacterial supernatant was prepared from MG1655 cells carrying pA-TP and incubated in M9 medium supplemented with the indicated carbon sources. Subsequently, the supernatant was concentrated using a centrifugal filter (Cat. No. UFC901008; Millipore), and the total fraction concentration was measured using the Bradford method. The concentrated supernatant (0.1 mg/mL) was then added to SW620, CT26, MC38, 4T1, and LLC1 cells cultured in 96-well plates for 48 h at 37 °C in a humidified chamber with 5% CO_2_. To assess cancer cell viability in response to TP exposure, MTT reagent was added to each well, followed by a 2-hour incubation at 37 °C to allow for formazan formation. Dimethyl sulfoxide (100 µL) was added to the plate and then resuspended to determine the dissolution of the purple crystals. A spectrophotometer (VLBL00D0; Thermo Fisher Scientific) was used to measure the OD of each well at 570 nm.

### Assessment of glucose and fumarate metabolites in vivo

To measure the relative nutrient ratios, liver and tumor tissues were collected from CT26-bearing mice when tumor volumes reached 100–150 mm³, before bacterial injection. Glucose [37] (Cat. No. ab169559; Abcam) and fumarate [38] (Cat. No. ab102516; Abcam) were quantified using colorimetric assay kits according to the manufacturer’s instructions. Freshly harvested tissues were lysed, and metabolite levels were determined from standard curves to obtain the number of nanomoles of each metabolite per gram of tissue. The glucose-to-fumarate ratio was calculated by dividing the glucose concentration by the fumarate concentration in each tissue, whereas the fumarate-to-glucose ratio was calculated by dividing the fumarate concentration by the glucose concentration.

### Bacterial RNA, cDNA library preparation, and quantitative polymerase chain reaction

Total RNA was extracted from in vitro-cultured *E. coli* and mouse organs infected with *E. coli* (the liver, spleen, and tumor) using a Monarch^®^ Total RNA Miniprep Kit (Cat. No. T2010S; New England Biolabs^®^). cDNA was synthesized from 1 to 5 µg of total RNA using reverse transcriptase with random hexamer primers (TOPscript™ cDNA Synthesis Kit, Cat. No. EZ005S; Enzynomics).

mRNA levels were quantified using quantitative polymerase chain reaction (qPCR) on a Rotor-Gene Q real-time PCR system (R072010103; Qiagen) with TOPreal™ qPCR 2× PreMIX (Cat. No. RT500S; Enzynomics). The expression levels of *dcuS*, *aspA*, *dcuB*, *frdA*, and *dctA* were normalized to those of the 16 S rRNA. The primers used in the experiments are listed in Supplementary Table S2. Each experiment was performed in triplicate.

### Statistical analysis

Data were analyzed using GraphPad Prism v.10.0.3 (GraphPad Software). Differences between the mean values of the two groups were analyzed using an unpaired two-tailed Student’s *t*-test. Two-way analysis of variance (ANOVA) was used for the time-course studies. Survival rates were determined using the log-rank (Mantel–Cox) test and are shown by Kaplan–Meier curves. Statistical significance was set at $\:P\:<0.05$.

## Results

### Nutrient heterogeneity in the TME drives the tumor-specific upregulation of *E. coliaspA* expression during the adaptive response

**Fig. 1:**
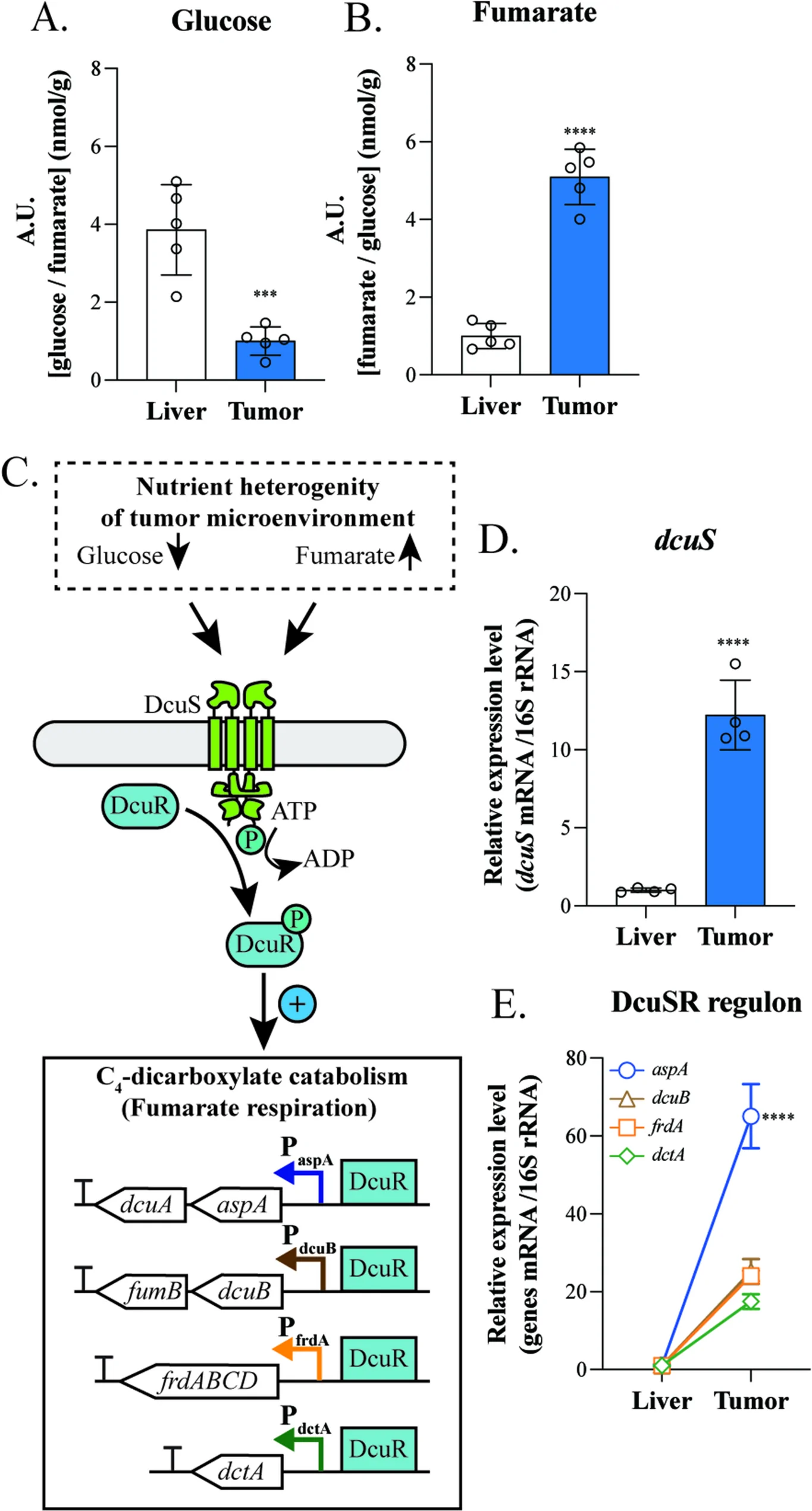
Nutrient heterogeneity in the CT26 tumor-microenvironment (TME) and the adaptive response of *Escherichia coli. *(**A**–**E**) BALB/c mice were subcutaneously implanted with 1 × 10^6^ CT26 colon carcinoma cells in the right thigh. (**A** and **B**) The nutrient ratio was measured in the indicated tissue (the white and blue bars represent the liver and tumor, respectively) collected when the tumor size reached 100–150 mm^3^ before injecting the bacteria into the mice. (**A**) The glucose-to-fumarate ratio, as determined by dividing the glucose concentration (nmol/g) by the fumarate concentration (nmol/g) in the indicated tissue. Data represent mean ± SD of n = 5 biological replicates. (**B**) The fumarate-to-glucose ratio, as determined by dividing the fumarate concentration (nmol/g) by the glucose concentration (nmol/g) in the indicated tissue. Data represent mean ± SD of n = 5 biological replicates. (**C**) Representation of the *E. coli* DcuSR two-component signal transduction system that responds to the presence of external fumarate and induces the expression of genes for its utilization. (**D** and **E**) CT26 tumor-bearing BALB/c mice were intravenously injected with wild-type MG1655 when the tumor size reached 100 mm^3^. (**D**) Relative gene expression levels of *dcuS*, as measured at 3 days post-bacterial injection (dpi) in the indicated tissue (white and blue bars represent *E. coli* gene expression in the liver and tumor, respectively). Data represent mean ± SD of n = 4 biological replicates. (**E**) Relative expression levels of DcuSR regulon genes measured at 3 dpi in the indicated tissues. Data represent mean ± SD of n = 4 biological replicates. ***P < 0.001, ****P < 0.0001; Unpaired two-tailed Student’s t-test for (**A**), (**B**), (**D**), and (**E**) (*aspA* vs. other genes within tumor)

We investigated nutrient heterogeneity to determine whether the TME provided a suitable environment for *E. coli* sensing, focusing on glucose depletion and fumarate accumulation in normal (e.g., the liver) and tumor tissues in CT26 tumor-bearing mice. The glucose-to-fumarate ratio was > 3-fold higher in the liver than in the tumor, whereas the fumarate-to-glucose ratio was > 5-fold lower in the liver than in the tumor (Fig. 1A and B). Moreover, in other major organs also, the glucose-to-fumarate ratio was higher than that in the tumor, and the fumarate-to-glucose ratio was lower than that in the tumor (Supplementary Fig. S1). These differences prompted us to explore whether *E. coli* responds to external carbon sources through its biological systems (Fig. 1C). The relative expression of *dcuS* was 10-fold higher in tumors than in the liver, prompting further investigation into the regulation of the DcuSR regulon within the TME (Fig. 1D). The expression of *aspA*, a gene within the DcuSR regulon, was remarkably higher in tumor tissues than in the normal liver tissues, with expression levels increasing approximately 65-fold. The upregulation was > 3-fold higher than that of any other gene within the DcuSR regulon in the TME (Fig. 1E), indicating that *aspA* was the most highly upregulated gene within the DcuSR regulon in tumor tissues. These findings indicate that the *aspA* promoter (P_aspA_) may serve as a metabolically responsive auto-inducible switch for anticancer agent tumor-specific expression.

### Carbon source–dependent *aspA* promoter activation

**Fig. 2:**
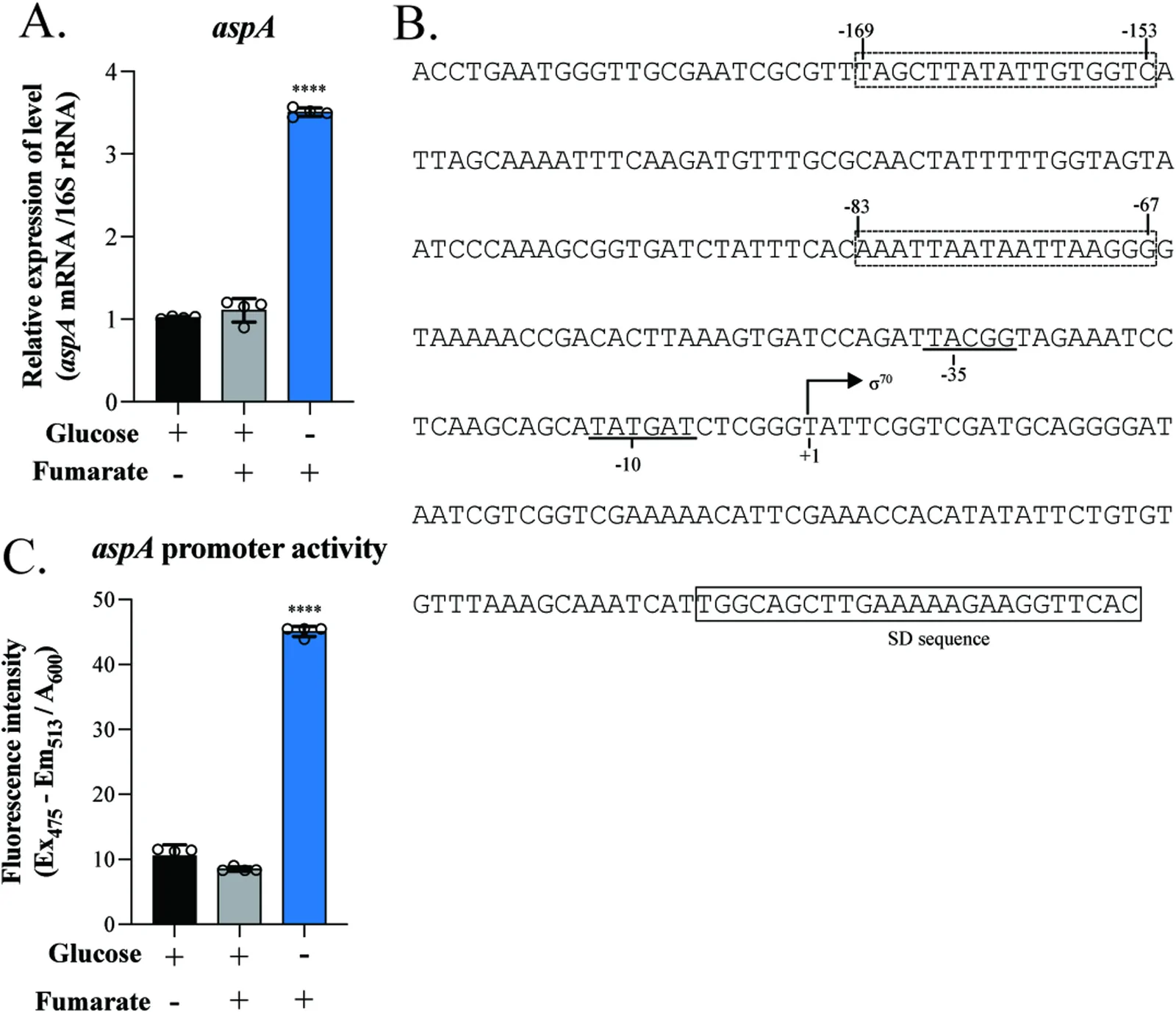
Assessment of *aspA* promoter (P_aspA_) activity under TME-like nutrient availability. (**A** and **C**) Bacteria were cultured in M9 medium supplemented with the indicated carbon source for 5 h in an anaerobic bag. (**A**) Relative gene expression levels of *aspA*, as measured after 5 h of incubation at the indicated culture conditions [black, glucose alone; gray, glucose + fumarate; blue, fumarate alone]. Data represent mean ± SD of n = 4 biological replicates (**B**) Representation of the promoter region of *aspA* (P_aspA_); dashed boxes indicate the C4-dicarboxylate-inducible DcuR-dependent activation site whereas solid boxes represent the ribosome-binding site. Solid underlines indicate the − 35 to − 10 RNA polymerase-binding region. (**C**) P_aspA_ activity was measured using an unstable variant of green fluorescent protein (gfpOVA), which was expressed by *E. coli* carrying pA-gfpOVA (MDG001) [black, glucose alone; gray, glucose + fumarate; blue, fumarate alone]. Fluorescence was detected after 5 h of incubation under the indicated culture conditions, using excitation and emission wavelengths of 488 and 513 nm, respectively. The measured fluorescence was normalized to the absorbance at 600 nm. Data represent mean ± SD of n = 4 biological replicates. ****P < 0.0001; Unpaired two-tailed Student’s t-test for (**A**) and (**C**)

We assessed whether the P_aspA_ is activated based on the availability of external carbon sources. First, wild-type *E. coli* was cultured in an anaerobic bag in M9 medium supplemented with the indicated carbon source for 5 h, based on the literature [35, 36]. Following incubation, we measured the relative expression of *aspA*. The expression of *aspA* was upregulated by > 3-fold in the presence of fumarate than in the presence of glucose (Fig. 2A). To confirm the functionality of P_aspA_ as a tumor-specific switch for anticancer agent delivery, we analyzed the P_aspA_ region to evaluate promoter activity [31, 34, 37]. The promoter was primarily regulated by phosphorylated DcuR, which bound to two regions located at − 83 to − 67 and − 169 to − 153 upstream of the *aspA* transcriptional start site (Fig. 2B). A gene reporter system was constructed using the unstable GFP variant *gfpOVA*, which was cloned downstream of P_aspA_ in pBR322 to generate pA-gfpOVA. *E. coli* transformed with this plasmid was used to monitor P_aspA_ activity. The fluorescence intensity was determined during in vitro growth in the aforementioned medium. Of note, glucose concentration in the tumor tissues, measured using colorimetric assay kits according to the manufacturer’s instructions, was 17.3 nmol/g and that of fumarate was 1150 nmol/g. The fluorescence intensity was measured at fumarate concentrations in the 0.1–20 mM range. The maximum fluorescence intensity was noted at a fumarate concentration of 20 mM (data not shown). The fluorescence intensity was > 4-fold higher in the fumarate medium than in the glucose medium (Fig. 2C and Supplementary Fig. S2).

### Cytotoxic activity of psp-TGFα-PE38 expressed via the *aspA* promoter

**Fig. 3:**
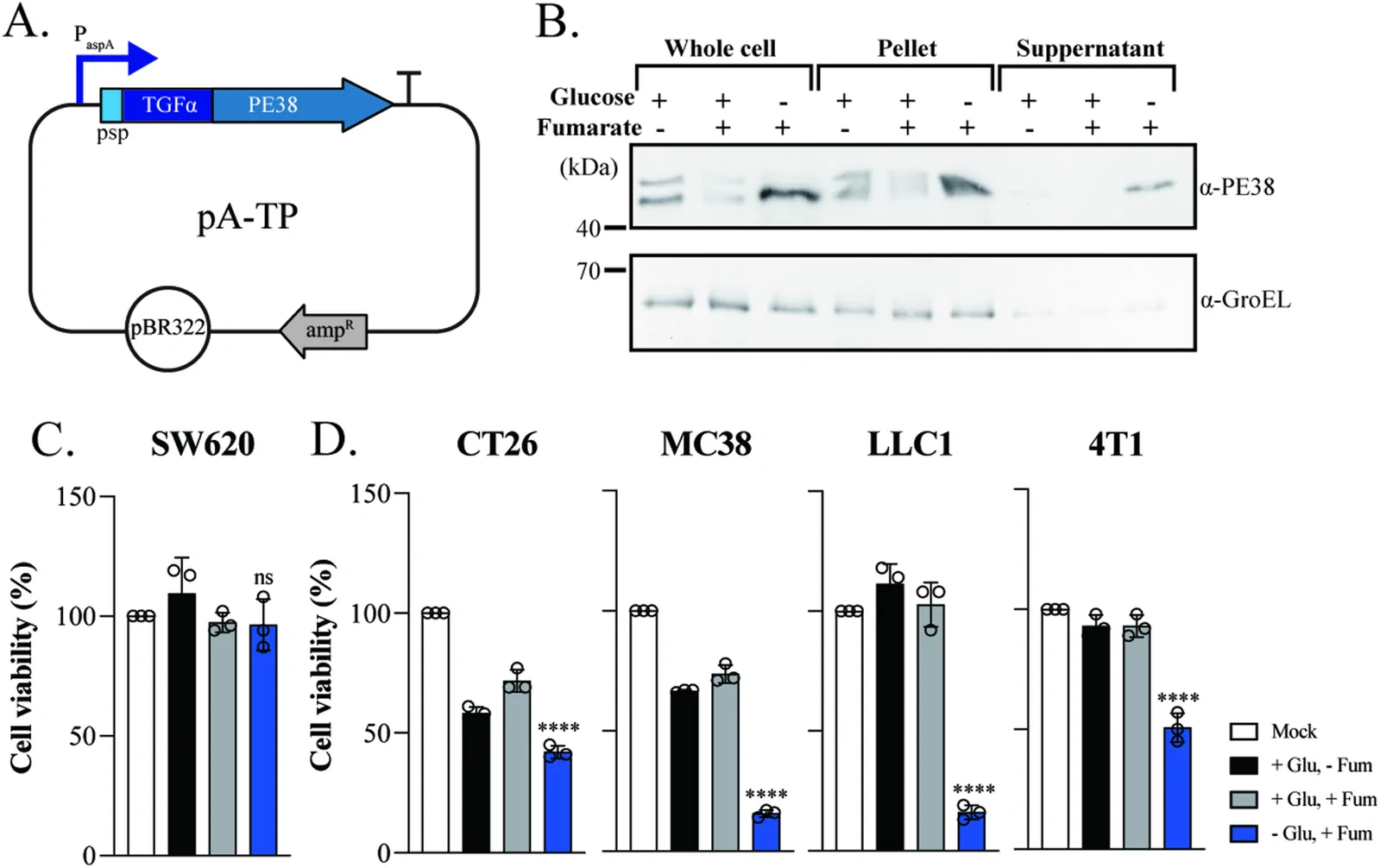
Selective cytotoxicity of the immunotoxin psp-TGFα-PE38 (TP), controlled by the *aspA* promoter (P_aspA_), against EGFR-overexpressing cancer cells. (**A**) A schematic of the plasmid (pA-TP) carrying psp-TGFα-PE38 (TP) controlled by P_aspA_. Escherichia coli carrying pA-TP (MDG003) was cultured in M9 medium supplemented with the indicated carbon source for 24 h in an anaerobic bag. (**B**) Expression and secretion of TP from MDG003 were detected via western blot analysis. MDG003 was cultured anaerobically in M9 medium supplemented with the indicated carbon sources (+Glu/-Fum; +Glu/+Fum; -Glu/+Fum) for 24 h. After incubation, the cultures were fractionated into whole-cell lysate, pellet, and supernatant. The supernatants were clarified via centrifugation and filtration. GroEL was used as a cytosolic marker to assess whether TP detected in the culture supernatant was actively secreted or passively released due to bacterial lysis. (**C** and **D**) Cytotoxic activity of TP within the supernatant from MDG003 was measured using the MTT assay in the indicated cell lines. MDG003 was cultured anaerobically in M9 medium supplemented with glucose and/or fumarate (+Glu/-Fum; +Glu/+Fum; -Glu/+Fum) as indicated, and clarified culture supernatants were applied to cells. Cell viability was calculated using the phosphate-buffered saline (PBS)-treated control (100%). (**C**) SW620 cells were used as an EGFR-negative control cell line. (**D**) CT26, MC38, LLC1, and 4T1 cells were used as EGFR-positive cell lines. Data represent mean ± SD of n = 3 biological replicates. ****P < 0.0001; one-way ANOVA for (**C**) and (**D**) with Dunnett’s comparisons test (-Glu/+Fum vs. other incubated conditions)

Next, we tested whether P_aspA_ could drive the expression of anticancer agents. We selected a recombinant immunotoxin composed of transforming growth factor alpha (TGFα) and modified *Pseudomonas* exotoxin A (PE38) as a model therapeutic payload, with the psp signal peptide fused in-frame to the N-terminal of TGFα-PE38 to facilitate secretion [39–41]. Therefore, we cloned psp-TGFα-PE38 (TP) under the control of P_aspA_ in pBR322 to generate pA-TP (Fig. 3A). Subsequently, we observed whether TP was successfully controlled by P_aspA_ under specific nutrient conditions similar to those in TME. TP was efficiently overexpressed with fumarate supplementation compared with its expression under glucose supplementation, supporting the functionality of P_aspA_ as a switch for the tumor-specific expression of TP (Fig. 3B).

To evaluate the selective cytotoxicity of TP expressed by P_aspA_, MTT assay was performed using the supernatant from *E. coli* cultures carrying pA-TP supplemented with glucose or fumarate. The assay was applied to CT26 and MC38 murine colon cancer cell lines, LLC1 murine lung cancer, and 4T1 murine breast cancer cells, all of which exhibit high levels of epidermal growth factor receptor (EGFR) expression [42, 43], compared with that in SW620 human colon cancer cells, which express low levels of EGFR [44]. The cytotoxic effect increased in proportion to the fumarate supplementation of the supernatant when 0.1 mg of the supernatant fraction was administered to tumor cells for 48 h, except for SW620 cells, which express low levels of EGFR (Fig. 3C). The maximum cytotoxicity of TP was observed in LLC1, a tumor cell line that overexpresses EGFR, showing approximately a 90% increase in cytotoxicity relative to the mock group (Fig. 3D). These results indicate that P_aspA_ enables the selective expression of the anticancer agent TP under TME-like conditions while preserving its cytotoxic effect against EGFR-overexpressing tumor cells.

### Tumor-specific TP expression via P_aspA_ suppressed tumor growth

**Fig. 4:**
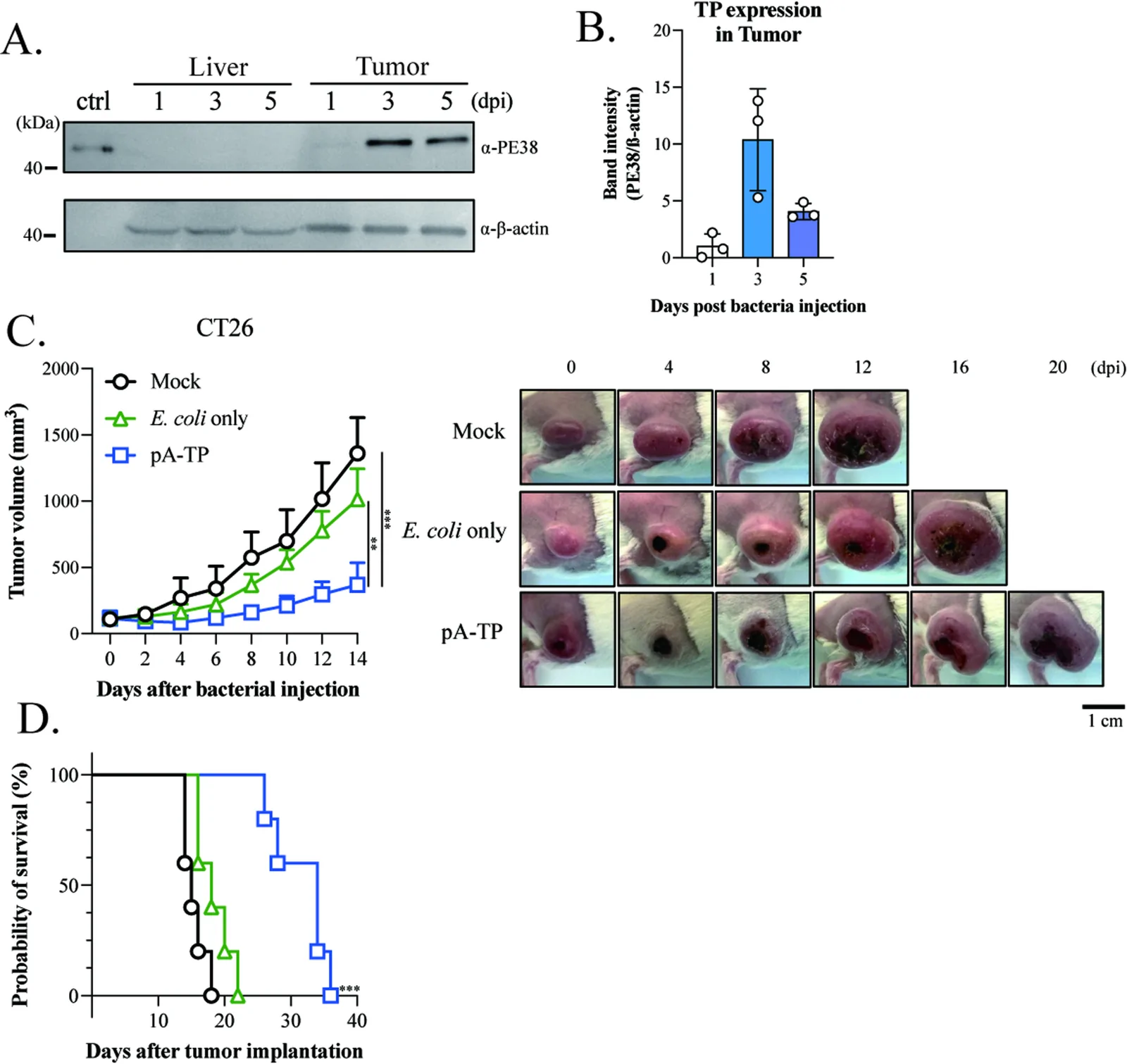
Anticancer effect of TP, driven by P_aspA_-mediated tumor-specific regulation in *Escherichia coli*, on CT26 colon carcinoma-grafted BALB/c mice. (**A**–**D**) BALB/c mice were subcutaneously implanted with 1 × 10^6^ CT26 colon carcinoma cells in the right thigh. (**A**) To assess the selective expression of TP by P_aspA_ in vivo, tumor-bearing mice were intravenously injected with *E. coli* carrying pA-TP (MDG003) (n = 3 per group; 10^8^ CFU/mouse) when tumor size approached approximately 100–150 mm^3^. TP was extracted by homogenizing the tissue in RIPA lysis buffer. The lysates were centrifuged and filtered. The in vivo expression of TP was determined through western blot analysis at 1, 3, and 5 dpi. Supernatant collected from a previous in vitro experiment under fumarate-induction conditions was used as control. (**B**) Quantitative analysis of band intensity using ImageJ software. *P < 0.1; Unpaired two-tailed Student’s t-test for (**B**) (Day 1 vs. other days). (**C**–**D**) CT26-bearing BALB/c mice were treated with PBS (Mock; black line circle), wild-type *E. coli* (*E. coli* only; green line triangle), or MDG003 (pA-TP; blue line square) by intravenous injection at a dose of 1 × 10^8^ CFU/mouse when tumor size approached approximately 100–150 mm^3^. (**C**) Tumor volumes (left) were measured every 2 days following bacterial injection until they reached > 1500 mm^3^. Data represent mean ± SD of n = 5 biological replicates. Representative images (right) show CT26 carcinoma progression in each group. (**D**) Survival of CT26-grafted mice was monitored until death when tumor volume exceeded 1500 mm^3^. Data represent mean ± SD of n = 5 biological replicates. The survival of CT26 tumor-bearing mice was determined using Kaplan–Meier curves. **P < 0.01, ***P < 0.001; Mixed-effects model (REML) for (**C**) with Geisser–Greenhouse correction and Holm–Šídák’s multiple comparisons test (*E. coli* pA-TP vs. other group at each time point); Log-rank (Mantel–Cox) test for (**D**) using Kaplan–Meier survival curves (*E. coli* pA-TP vs. *E. coli* only)

The role of P_aspA_ as a metabolically responsive promoter that enables tumor-specific expression of the immunotoxin TP was initially validated in vitro. Subsequently, we evaluated its ability to drive selective TP expression in the TME and minimize off-target expression in normal tissues in vivo. To this end, CT26 tumor-bearing mice were intravenously administered *E. coli* carrying pA-TP. Western blot analysis of TP expression in tumor and liver tissue showed that TP was detectable in tumors, beginning day 1; it increased by more than 10-fold on day 3 and then declined on day 5; however, no TP expression was detected in the liver tissue (Fig. 4A, B, and Supplementary Fig. S3). The results revealed that TP is selectively expressed within the TME under the control of P_aspA_. Furthermore, tumor volumes were used to evaluate the anti-tumor efficacy of this system. Mice treated with *E. coli* carrying pA-TP exhibited a 2.8-fold reduction in tumor growth compared with the mock treatment and a 2.1-fold reduction compared with wild-type *E. coli* (Fig. 4C, with images of all tumors presented in Supplementary Fig. S4). Consistent with the tumor suppression results, survival rate analysis indicated that treatment with *E. coli* carrying pA-TP prolonged the lifespan of tumor-bearing mice by up to 35 days (Fig. 4D). Although *E. coli* carrying pA-TP showed precise efficacy in delivering anticancer agents, the associated side effects of the bacteria should be carefully monitored. The body weight loss in the treated mice was < 6%, with a noticeable recovery after 4 dpi (Supplementary Fig. S5A). The bacterial loads in normal tissues were over 100,000-fold lower than those in tumor tissues after 3 days (Supplementary Fig. S5B). Correspondingly, serum IL-6 levels recovered significantly after 3 dpi compared with those observed after 1 dpi, indicating a reduction in systemic inflammatory responses (Supplementary Fig. S5C). To further assess systemic toxicity, we measured serum ALT, AST, and BUN levels after bacterial administration. ALT and AST levels were transiently increased after bacterial injection, with a maximum increase of approximately 3-fold, but returned to the normal range over time. BUN levels, although exhibiting inter-individual variations, remained within the normal range on day 1. These results suggest that bacterial administration caused a transient hepatic response without persistent liver injury or apparent renal dysfunction (Supplementary Fig. S3D-F).

## Discussion

Effective cancer therapy requires both potent antitumor activity and precise tumor selectivity to minimize systemic toxicity. Recent advances in tumor-responsive therapeutic platforms, including nanomedicine-based systems that respond to tumor-associated pH imbalance, metabolic alterations, or intracellular biochemical cues, have provided important strategies to improve the local activity of conventional chemotherapeutic agents within the TME [45, 46]. However, these platforms generally deliver a finite amount of preloaded therapeutic agents and do not enable continuous production of therapeutic proteins within tumor tissue. Bacterial cancer therapy offers a distinct strategy by harnessing engineered microbes that preferentially colonize the TME and can function as local production platforms for therapeutic payloads [5–7]. Recent synthetic biology approaches have further expanded this concept by engineering bacteria to respond to tumor-associated metabolic signals. For example, in a recent study, a kynurenine-responsive *S. enterica* system was developed, in which tumor-enriched kynurenine was used to control bacterial growth and enhance tumor-selective accumulation [47]. This strategy highlights the potential of using dysregulated tumor metabolism as an intrinsic cue to improve the tumor specificity of bacterial cancer therapy. However, this approach primarily focuses on metabolite-dependent control of bacterial growth and colonization. Studies specifically exploiting the metabolic features of the TME to achieve tumor-selective expression of anticancer agents are limited [6, 10]. In this study, we successfully developed and validated a TME-responsive gene expression system in *E. coli* by leveraging the distinctive metabolic characteristics of the TME as intrinsic cues for therapeutic gene expression.

We began by systematically characterizing the nutrient heterogeneity within the TME of CT26 tumor-bearing mice. Consistent with previous reports [11, 12, 14, 16, 22, 23], our metabolic analysis unequivocally showed significant glucose depletion and fumarate accumulation in tumor tissues compared with that in normal liver tissues (Fig. 1A and B). This distinct metabolic signature provided the foundational rationale for exploiting TME-specific cues. Based on these findings, we investigated the DcuSR regulon as a promising metabolic-responsive system (Fig. 1C) [26–29]. Crucially, the aspartase-encoding *aspA* gene exhibited the most pronounced tumor-specific upregulation (65-fold, Fig. 1D), positioning its promoter, P_aspA_, as an ideal candidate for a tumor-specific, auto-switch.

Our in vitro experiments confirmed robust activation of P_aspA_ under tumor-like metabolic conditions and minimal activity under normal metabolic conditions (Fig. 2). To translate this into a therapeutic application, we engineered pA-TP, encoding the recombinant immunotoxin TP under P_aspA_ control (Fig. 3A). TP comprises two functional domains designed for selective cytotoxicity: a TGFα moiety that binds the epidermal growth factor receptor (EGFR), which is frequently overexpressed in malignant tumors, and a truncated *Pseudomonas* exotoxin A (PE38) domain, which inhibits protein synthesis upon internalization, leading to cell death. Through this design, TP combines receptor–ligand targeting specificity with the potent cytotoxicity of bacterial toxins. Despite the therapeutic promise of immunotoxins, their systemic administration often results in off-target toxicity and limited efficacy, highlighting the need for localized delivery strategies. In this context, the tumor-responsive P_aspA_ system offers a strategy to localize TP expression within the tumor microenvironments.

In vitro assays showed that TP expression was precisely induced under tumor-like conditions, with minimal expression in normal metabolic environments (Fig. 3B). Furthermore, the secreted TP retained its EGFR–TGFα-mediated selective cytotoxicity against EGFR-overexpressing cancer cells (Fig. 3C and D), confirming its functional integrity. Although, the cytotoxicity observed in culture supernatants under conditions with low P_aspA_ activity may include basal cytotoxicity, which influenced cell line–dependent effects caused by bacterial supernatant components [48, 49]. The therapeutic relevance of the P_aspA_ system was further validated in vivo. Intravenous administration of *E. coli* carrying pA-TP into CT26 tumor-bearing mice resulted in selective TP expression within tumors (Fig. 4A and B). This selective expression led to remarkable tumor regression and a substantial prolongation of survival in treated mice (Fig. 4C and D). These compelling in vivo results confirm that the P_aspA_ system effectively leverages TME-specific metabolic conditions to enable the selective expression of therapeutic proteins. Our approach enhanced on-target accuracy while significantly reducing the risk of off-target toxicity, thereby improving the overall efficacy of bacteria-mediated anticancer therapy.

Despite these promising outcomes, complete tumor elimination was not achieved, likely because of the inherent challenge of stable plasmid maintenance in bacteria in vivo. The plasmid-bearing fraction of tumor-associated bacteria gradually decreased over time, from approximately 60% on 1 dpi to 1% on 5 dpi (Supplementary Fig. S6). Although partial loss of the plasmid led to reduced therapeutic gene expression, bacterial component stimulated immune activation in tumor, which sustained the anticancer effect. However, this limitation was overcome in bacterial cancer therapy. Future strategies to overcome this critical issue include employing essential gene complementation systems, such as *asd* and *glmS*, which can significantly promote stable plasmid inheritance and thus enhance the long-term therapeutic potential of the P_aspA_ system. Additionally, recent advances in synthetic biology and nanomedicine provide opportunities to enhance the therapeutic efficacy of bacterial cancer therapy. For example, tumor-targeting bacteria can be combined with nanomedicine-based strategies, including hitchhiking nanoparticles on bacterial surfaces, to improve intratumoral delivery, penetration, and local therapeutic activity [50]. Engineered bacteria can also be used as intratumoral delivery platforms for immunomodulatory molecules, which can remodel the tumor microenvironment and enhance antitumor immune responses. Furthermore, combination strategies with immune checkpoint inhibitors may potentiate therapeutic efficacy by exploiting bacteria-induced immune activation [51]. In this context, the P_aspA_ expression system developed in this study may also be applicable to combination strategies for bacterial cancer therapy in the future. Because P_aspA_ functions as a tumor-associated metabolic switch, it can be used to control the expression of diverse therapeutic payloads, including immunomodulators, cytokines, enzymes, nanobodies, and other protein-based anticancer agents. Thus, the P_aspA_ system may provide an additional regulatory layer that improves the precision and safety of future bacteria-based combination therapies while expanding the therapeutic potential of tumor-targeting bacterial platforms.

With the ongoing advancements in synthetic biology and genetic engineering, we anticipate that the P_aspA_ system will evolve into a versatile and highly reliable platform. Its auto-inducible, tumor-specific nature makes it particularly promising for integration with other therapeutic strategies, potentially paving the way for more effective and precisely targeted cancer treatments.

## Conclusion

We developed a tumor-responsive gene expression system based on the *aspA* promoter that enables tumor-selective expression of anticancer agents by exploiting metabolic features of the TME. Our findings demonstrate that TME-associated nutrient heterogeneity, particularly glucose depletion and fumarate accumulation, can be harnessed as intrinsic regulatory cues for controlling therapeutic gene expression in bacteria (Graphic abstract). This strategy addresses a critical limitation in bacterial cancer therapy by enabling spatially restricted expression of therapeutic payloads, thereby improving targeting precision and reducing the risk of off-target toxicity. Importantly, the P_aspA_-based system represents a modular and adaptable platform that can be extended to a wide range of therapeutic protein beyond immunotoxins. Overall, our study provides a new framework for leveraging endogenous tumor-associated signals to achieve precise and context-dependent control of therapeutic gene expression in bacterial cancer therapy. 

## Supplementary Information

Below is the link to the electronic supplementary material.

Supplementary Material 1

## Data Availability

The raw data supporting the conclusions of this article will be made available by the authors upon reasonable request.

## Abbreviations

ANOVA: Analysis of variance
dpi: days post-injection
EGFR: Epidermal growth factor receptor
PBS: Phosphate-buffered saline
qPCR: quantitative Polymerase Chain Reaction
TGFα: Transforming growth factor alpha
TME: Tumor microenvironment
