# Supplementary material for: aspA promoter as a tumor microenvironment-responsive autoswitch for anticancer agent expression in Escherichia coli
Source: J Biol Eng. 2026 Jul 31;20:156. doi: 10.1186/s13036-026-00743-9 (PMC13715239; doi:10.1186/s13036-026-00743-9)
Supplement: Supplementary file 1 [file 13036_2026_743_MOESM1_ESM.docx]

| Construction | Name and direction | | Sequence (5′ ® 3′) | Reference |
| --- | --- | --- | --- | --- |
| pA-gfpOVA | P_aspA_-insert-FW | AAGCATGCTACCTGAATGGGTTGCGAAT | | This study |
|  | P_aspA_-insert-RV | AATCTAGACGCTCTCAGAGTGTGAACAC | | This study |
|  | Seq-GFP-FW | TCTCCACTGACAGAAAATTTGTGC | | This study |
| pA-TP | TP-vector-FW | GCGCGAGGACCTGAAGTAACTGCAGCCAAGCTTGGCT | | This study |
|  | TP-vector-RV | CTGATCTTTTCTTCATCTTCAAACCCATATGTATATCTCCTTCTTAAATCTAGACGCTCT | | This study |
|  | P_aspA_-insert-FW | AGAGCGTCTAGATTTAAGAAGGAGATATACATATGGGTTTGAAGATGAAGAAAAGATCAG | | This study |
|  | P_aspA_-insert-RV | AGCCAAGCTTGGCTGCAGTTACTTCAGGTCCTCGCGC | | This study |
|  | Seq-TP-FW | GCGACTCCTGCATTAGGAAGCAGC | | This study |

**Supplementary Table S1. List of primers for plasmid construction**

*FW: Froward primer

* RV: Reverse primer

**Supplementary Table S2. Primer sets for qRT-PCR**

| qRT-PCR primer description | | Sequence (5′ ® 3′) | Reference |
| --- | --- | --- | --- |
| dcuS | FW primer | TGCGGTACTGTTCTCGGTGC | This study |
|  | RV primer | CACTGCCAGTGCCTTGTTGG | This study |
| aspA | FW primer | ATGTCAAACA ACATTCGTAT | This study |
|  | RV primer | AGTTTTCAATCGCTCTCAGA | This study |
| dcuB | FW primer | AGAAAGGGTGGTATCGCGCT | This study |
|  | RV primer | CCACCACCGCAATGATAACC | This study |
| frdA | FW primer | GCGCAGGCAAATCCGAATGC | This study |
|  | RV primer | CATGATCCTGCGCGACAGCG | This study |
| dctA | FW primer | GGTCCTGACAGCGATAGCCA | This study |
|  | RV primer | CCGCAATGCCCGTTACGACG | This study |
| 16S rRNA | FW primer | CATGCCGCGTGTATGAAGAA | 42 |
|  | RV primer | CGGGTAACGTCAATGAGCAAA | 42 |

*FW: Froward primer

* RV: Reverse primer

**Supplementary Figure 1. Relative carbon source ratio in different organs of CT26 tumor-bearing mice**

**(A and B)** BALB/c mice were subcutaneously implanted with 1 × 10^6^ CT26 colon carcinoma cells in the right thigh. **(A and B)** The relative nutrient content was measured in the indicated tissues collected when the tumor size reached 100–150 mm^3^ before injecting the bacteria into the mice. **(A)** The glucose-to-fumarate ratios, determined by dividing the glucose concentration (nmol/g) by the fumarate concentration (nmol/g) in the indicated tissue. Data represent mean ± SD values for n = 5 biological replicates. **(B)** The fumarate-to-glucose ratios, determined by dividing the fumarate concentration (nmol/g) by the glucose concentration (nmol/g) in the indicated tissue. Data represent mean ± SD values for n = 5 biological replicates. *****P* < 0.0001; one-way ANOVA with Dunnett’s comparison test (tumor vs. other organ).

**Supplementary Figure S2. Growth environment-dependent P_aspA_ activity.**

**(A and B)** *E. coli* carrying pA-gfpOVA were cultured in M9 medium supplemented with the indicated carbon source in an anaerobic bag. **(A)** Bacterial growth was monitored by measuring the absorbance at 600 nm hourly. **(B)** *aspA* promoter activity was evaluated by utilizing an unstable variant of green fluorescent protein (gfpOVA), with fluorescence detected at excitation and emission wavelengths of 488 nm and 513 nm, respectively. Fluorescence intensity was normalized to absorbance at 600 nm.

**Supplementary Figure S3. Western blot images of TP expression in all individual liver and tumor samples from CT26 tumor-bearing mice**

BALB/c mice were subcutaneously implanted with 1 × 10^6^ CT26 colon carcinoma cells on the right thigh. CT26-bearing BALB/c mice were intravenously injected MDG003 by at a dose of 1 × 10^8^ colony-forming unit/mouse when the tumor volume was 100–150 mm^3^. TP was extracted from the liver and tumor tissues by homogenizing the tissue in RIPA lysis buffer. The lysates were centrifuged and filtered. The *in vivo* expression of TP was determined through western blot analysis on 1, 3, and 5 dpi. Supernatant collected from a previous *in vitro* experiment under fumarate-induction conditions was used as control. The red arrows indicate the PE38 band.

**Supplementary Figure S4. Complete set of tumor-bearing mouse images expanded from those shown in Figure 4B.** BALB/c mice were subcutaneously implanted with 1 × 10^6^ CT26 colon carcinoma cells on the right thigh. CT26 tumor-bearing mice were intravenously injected with PBS (Mock; black line), wild-type *E. coli* (*E. coli* only; green line), or MDG003 (pA-TP; blue line) (n = 5 per group; 10^8^ CFU/mouse) when the tumor size was 100–150 mm^3^.

**Supplementary Figure S5. Assessment of the host response to bacterial injection**

**(A–F)** BALB/c mice were subcutaneously implanted with 1 × 10^6^ CT26 colon carcinoma cells on the right thigh. CT26-bearing BALB/c mice were intravenously injected phosphate-buffered saline (Mock; black line circle), wild-type *Escherichia coli* (*E. coli* only; green line triangle), or MDG003 (pA-TP; blue line square) at a dose of 1 × 10^8^ colony-forming unit/mouse when the tumor size was 100–150 mm^3^. **(A)** Changes in the body weight of mice grafted with CT26, measured every 2 days after bacterial injection. **(B)** MDG003 levels on 1, 3, 5, and 30 dpi in the indicated tissues after MDG003 injection. **(C)** Serum IL-6 levels on 1, 3, and 5 dpi after MDG003 injection. **(D–F)** Serum ALT **(D)**, AST **(E)**, and BUN **(F)** levels on 1, 3, 5, and 30 dpi after MDG003 injection. Normal range values: ALT: 19.1–32.9 U/L; AST: 123.5–153.7 U/L; BUN: 18.3–27.6 mg/dL. The yellow-shaded areas show the normal range. ns < 0.1, **P* < 0.01, ****P* < 0.001, *****P* < 0.0001; one-way ANOVA multiple comparisons with Dunnett’s comparison test (normal vs. other dpi).

**Supplementary Figure S6. Assessment of the plasmid stability *in vivo*.**

BALB/c mice were subcutaneously implanted with 1 × 10^6^ CT26 colon carcinoma cells on the thigh. To assess the toxicity of bacterial injection, CT26-bearing BALB/c mice were treated with MDG003 by intravenous injection at a dose of 1 × 10^8^ CFU/mouse when the tumor size was 100–150 mm^3^. Bacterial counts were measured at 1, 3, and 5 dpi in the tumor tissue. To assess plasmid stability, recovered bacteria were plated on both non-selective LB agar and antibiotic-containing LB agar. Plasmid-bearing fraction was calculated as the percentage of CFUs grown on amp-containing plates relative to total CFUs grown on non-selective LB plates.
